# Heart Rate Variability Change Before and After Hemodialysis is Associated with Overall and Cardiovascular Mortality in Hemodialysis

**DOI:** 10.1038/srep20597

**Published:** 2016-02-08

**Authors:** Szu-Chia Chen, Jiun-Chi Huang, Yi-Chun Tsai, R. N. Hsiu-Chin Mai, R. N. Jui-Hsin Chen, Po-Lin Kuo, Jer-Ming Chang, Shang-Jyh Hwang, Hung-Chun Chen

**Affiliations:** 1 Graduate Institute of Clinical Medicine, College of Medicine, Kaohsiung Medical University, Kaohsiung, Taiwan; 2 Division of Nephrology, Department of Internal Medicine, Kaohsiung Medical University Hospital, Kaohsiung Medical University, Kaohsiung, Taiwan; 3 Department of Internal Medicine, Kaohsiung Municipal Hsiao-Kang Hospital, Kaohsiung Medical University, Kaohsiung, Taiwan; 4 Department of Nursing, Kaohsiung Municipal Hsiao-Kang Hospital, Kaohsiung Medical University, Kaohsiung, Taiwan; 5 Faculty of Medicine, College of Medicine, Kaohsiung Medical University, Kaohsiung, Taiwan; 6 Institute of Medical Science and Technology, National Sun Yat-Sen University, Kaohsiung, Taiwan; 7 Department of Internal Medicine, Kaohsiung Municipal Cijin Hospital (Operated by Kaohsiung Medical University), Kaohsiung, Taiwan

## Abstract

Low heart rate variability (HRV) has been recognized to correlate with adverse cardiovascular (CV) outcomes in hemodialysis (HD) patients. It has been reported that HRV might be improved after HD, but whether the improved HRV after HD predicts a better CV prognosis remains to be determined. This study examined the ability of the change in HRV before and after HD in predicting overall and CV mortality in HD patients. This study enrolled 182 patients under maintenance HD. HRV was examined to assess changes before and after HD. The change in HRV (ΔHRV) was defined as post-HD HRV minus pre-HD HRV. During a median follow-up period of 35.2 months, 29 deaths (15.9%) were recorded. Multivariate analysis showed that decreased ΔLF% was associated with increased overall (hazard ratios [HR], 0.978; 95% confidence interval [CI], 0.961–0.996; *p* = 0.019) and CV mortality (HR, 0.941; 95% CI, 0.914–0.970; *p* < 0.001), respectively. Moreover, adding ΔLF% to a clinical model provided an additional benefit in the prediction of overall (*p* = 0.002) and CV mortality (*p* < 0.001). HRV change before and after HD (ΔHRV) is an useful clinical marker, and it is stronger than HRV before HD in predicting overall and CV mortality.

End-stage renal disease (ESRD) is a global public health burden bearing high morbidity and mortality, and cardiovascular (CV) disease is a major cause of mortality in hemodialysis (HD) patients^1^,^2^. In HD patients, CV autonomic neuropathy and the related risk of arrythmia may partially explain the observed high rate of CV mortality besides the traditional risk factors, including hypertension, diabetes, and dyslipidemia^3^,^4^.

CV autonomic neuropathy can be evaluated by heart rate variability (HRV), a measure of variations in heart rate^5^. In practice, it is defined as variations of both instantaneous heart rate and R-R intervals of electrocardiogram, and may provide a simple and noninvasive way to assess the activities of the autonomic nervous system^5^. Abnormal HRV primarily reflects the dys-regulation between the sympathetic and parasympathetic nervous system. Frequency-domain analysis of HRV has gained popularity with broad application as a functional indicator of the autonomic nervous system, for its non-invasiveness and easy accessibility.

A low HRV, indicating impaired autonomic function, has been noted in HD population^3^. Furthermore, reduced HRV has been shown to be associated with adverse CV outcomes and mortality in HD patients^3^,^4^, and HD itself may improve HRV^6^,^7^,^8^. Whether the improved HRV after HD foretells a better CV prognosis remains to be determined. Therefore, the aim of this study is to assess whether the change in HRV before and after HD is associated with overall and CV mortality in HD patients.

## Patients and Methods

### Study Patients and Design

The study was conducted in a regional hospital in southern Taiwan. All the maintenance HD patients in this hospital were included, except patients receiving HD at night shift. Totally, we enrolled 182 patients (81 males and 101 females) from May 2012 to July 2012. All the patients received HD three times per week, and each HD session was performed for 3.5–4.5 hours with a blood flow rate of 250–300 mL/min and dialysate flow of 500 mL/min. Blood samples were taken before HD, and also after HD to calculate Kt/V. The study protocol was approved by the Institutional Review Board of Kaohsiung Medical University Hospital, and all participants provided written informed consent to participate in this study. The methods were carried out in accordance with the approved guidelines.

### Electrocardiogram signal processing

All recruited subjects received a short-term power spectral analysis of HRV. All measurements for spectral analysis were conducted in a quiet, temperature-controlled (28 °C) room. The procedure for HRV analysis was designed according to the standard method, and detailed procedures for HRV analysis have been reported previously^9^,^10^,^11^. A pericardial electrocardiogram (ECG) was taken for continuous 5 min with the patients laying quietly and breathing normally in the supine position for at least 10 min. The study patients received ECG examination before and after HD 30 minutes, respectively, during the day (between 8 a.m. and 5 p.m.). ECG signals were recorded using an HRV analyzer (SS1C, Enjoy Research, Taipei, Taiwan) with an analog-to-digital converter and sampling rate of 256 Hz. Digitized ECG signals were analyzed online and were simultaneously stored for off-line verification. Signal acquisition, storage, and processing were performed on a computer. The computer algorithm then identified each QRS complex and rejected each ventricular premature complex or noise according to its likelihood in a standard QRS template. Stationary R-R values were re-sampled and interpolated at a rate of 7.11 Hz to produce continuity in the time domain^10^.

### HRV frequency-domain analysis

Frequency-domain analysis was performed using a non-parametric method of fast Fourier transformation (FFT). The direct current component was deleted, and a Hamming window was used to attenuate the leakage effect^12^. For each time segment (288 s; 2048 data points), our algorithm estimated the power spectrum density based on FFT. The resulting power spectrum was corrected for attenuation resulting from the sampling and the Hamming window. The power spectrum was subsequently quantified into standard frequency-domain measurements as defined previously^5^, including very low frequency (VLF) (0.003–0.04 Hz), low frequency (LF) (0.04–0.15 Hz), high frequency (HF) (0.15–0.40 Hz) and LF/HF HRV. LF was normalized by the percentage of total power to detect the sympathetic influence on HRV (LF% = LF/(total power-VLF)*100). A similar procedure was applied to HF (HF% = HF/(total power-VLF)*100). All HRV parameters were logarithmically transformed to correct for the skewness of the distribution^5^.

### Collection of demographic, medical, and laboratory data

Demographic and medical data including age, gender and co-morbid conditions were obtained from medical records and interviews with patients. Laboratory data were measured from fasting blood samples using an autoanalyzer (Roche Diagnostics GmbH, D-68298 Mannheim COBAS Integra 400). Serum intact PTH (iPTH) concentration was evaluated using a commercially available two-sided immunoradiometric assay (CIS bio international, France). Kt/V was evaluated as a marker of dialysis efficiency and was determined according to the Daugirdas procedure^13^.

### Definition of overall and CV mortality

Overall and CV deaths were confirmed and ascertained from the medical records by 2 cardiologists, and disagreements were resolved through the adjudication of a third cardiologist. Patients were followed until death, and the remaining patients were followed until June 2015. Patients with no observed mortality were censored at the end of the follow-up.

### Statistical analysis

Statistical analysis was performed using SPSS 17.0 for windows (SPSS Inc. Chicago, USA). Data are expressed as percentages, mean ± standard deviation, or mean ± standard error of the mean for HRV parameters, or median (25^th^–75^th^ percentile) for duration of dialysis, triglyceride and iPTH. The differences between groups were checked by Chi-square test for categorical variables, by independent t-test for continuous variables with approximately-normal distribution, or by Mann-Whitney U test for continuous variables with skewed distribution. Paired t-test was used to compare HRV parameters before and after HD. The ΔHRV parameters were defined as HRV parameters measured after HD minus HRV parameters measured before HD. Multiple forward stepwise Cox proportional hazard analysis was used to identify the factors associated with overall and CV mortality. Survival curve for overall and CV mortality was derived using Cox-regression analysis. Incremental model performance was assessed using a change in the χ^2^ value. A difference was considered significant if the *p* value was less than 0.05.

## Results

The mean age of the 182 patients was 61.2 ± 11.3 years. The baseline characteristics in all patients were shown in Table 1.

### Change of HRV parameters before and after HD

Changes of HRV parameters before and after HD was shown in Table 2. In patients with sur vival, VLF (*p* < 0.001), LF% (*p* < 0.001) and LF/HF (*p* < 0.001) significantly increased after HD, and HF% (*p* = 0.006) decreased after HD, whereas no significant change of HRV parameters was noted in patients with mortality.

### Risk of overall mortality

The median and range of follow-up period was 35.2 (3.8–38) months for all patients. During the follow-up period, 29 deaths were recorded among these 182 patients (15.9%), including CV deaths (n = 11), malignancy (n = 4), infectious disease (n = 12), gastrointestinal bleeding (n = 1), and others (n = 1). Table 3 shows the predictors for overall mortality adjustment for age, sex, smoking history, duration of dialysis, history of diabetes, coronary artery disease and cerebrovascular disease, systolic blood pressure, albumin, triglyceride, total cholesterol, hemoglobin, creatinine, potassium, total calcium, phosphorous, iPTH, Kt/V, ultrafiltration rate, HRV parameters before HD and ΔHRV parameters. In the multivariate forward analysis, old age, decreased albumin, decreased Kt/V, increased ultrafiltration rate and decreased ΔLF% (hazard ratios [HR], 0.978; 95% confidence interval [CI], 0.961–0.996; *p* = 0.019) were independently associated with increased overall mortality. We had further performed the interactions between ΔLF % and age, albumin, Kt/V and ultrafiltration percentage with overall survival, and there were no statistical significances among the interaction terms.

**Figure 1:**
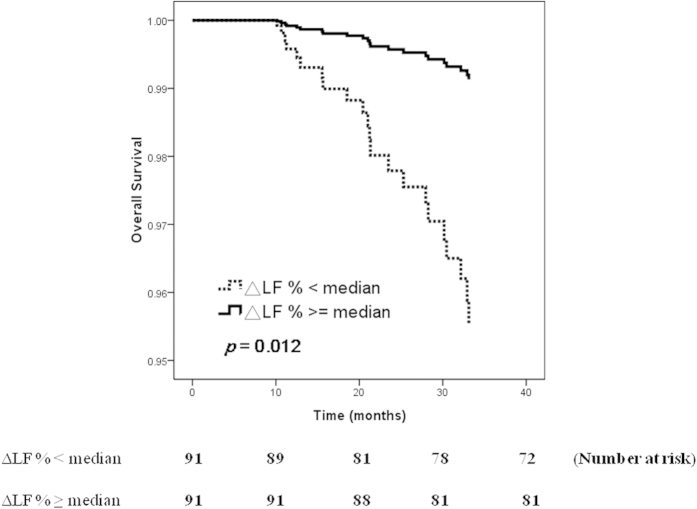
Adjusted overall survival curves in patients according to median level of ΔLF% (*p* = 0.012). Patients with ΔLF% < median had a worse overall survival than those with ΔLF% ≧ median.

Figure 1 illustrated the adjusted Cox regression survival curves [adjusted for age, sex, smoking history, duration of dialysis, history of diabetes, hypertension, coronary artery disease and cerebrovascular disease, systolic and diastolic blood pressure, albumin, fasting glucose, triglyceride, total cholesterol, hemoglobin, creatinine, potassium, total calcium, phosphorous, calcium-phosphorous (Ca × P) product, iPTH, Kt/V and ultrafiltration rate] for overall survival in patients according to median level of ΔLF% (5.1 nu). Patients with ΔLF% < median had a worse overall survival than those with ΔLF% ≧ median (HR, 0.192; 95% CI, 0.053–0.699; *p* = 0.012).

### Risk of CV mortality

The documented CV deaths during follow-up included heart failure (n = 4), myocardial infarction (n = 2), ventricular fibrillation (n = 2) and hemorrhagic stroke (n = 3). Table 3 also lists the predictors for CV mortality adjustment for demographic, clinical, biochemical factors, HRV parameters before HD and ΔHRV parameters. Multivariate forward analysis revealed that a history of diabetes, decreased hemoglobin and decreased ΔLF% (HR, 0.941; 95% CI, 0.914–0.970; *p* < 0.001) were significantly associated with increased CV mortality. We had further performed the interactions between ΔLF % and a history of diabetes and hemoglobin with CV survival, and there were no statistical significances among the interaction terms.

**Figure 2:**
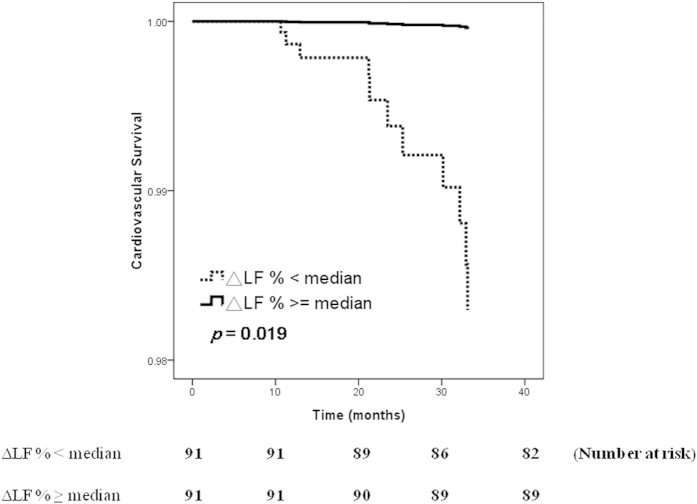
Adjusted cardiovascular survival curves in patients according to median level of ΔLF% (*p* = 0.019). Patients with ΔLF% < median had a worse cardiovascular survival than those with ΔLF% ≧ median.

Figure 2 illustrated the adjusted Cox regression survival curves (adjusted for demographic, clinical, biochemical factors) for CV survival in patients according to median level of ΔLF% (5.1 nu). Patients with ΔLF% < median had a worse CV survival than those with ΔLF% ≧ median (HR, 0.027; 95% CI, 0.001–0.550; *p* = 0.019).

### Incremental value of ΔLF% in relation to overall and CV mortality

We perform the analysis for the incremental value of ΔLF% in the outcomes prediction. The clinical model includes age, sex, smoking history, duration of dialysis, a history of diabetes, hypertension, coronary artery disease and cerebrovascular disease, systolic and diastolic blood pressure, albumin, fasting glucose, triglyceride, total cholesterol, hemoglobin, creatinine, potassium, total calcium, phosphorous, Ca × P product, iPTH, Kt/V and ultrafiltration rate. Adding ΔLF% to the clinical model provided an additional benefit in the prediction of overall mortality (χ^2^ = 47.55 to 57.10, *p* = 0.002). In addition, adding ΔLF% to the clinical model showed an additional benefit in the prediction of CV mortality (χ^2^ = 23.30 to 38.63, *p* < 0.001).

## Discussion

In the present study, we evaluated the relationship between ΔHRV parameters and overall/CV mortality in HD patients. We found that decreased ΔLF% were associated with increased overall and CV mortality. Furthermore, ΔLF% improved prediction for overall and CV mortality.

HRV is a non-invasive measure of autonomic nervous system, which reflects beat-to-beat variability in heart rate, and has been successfully applied in chronic dialysis patients^14^. HRV has been categorized into HF and LF ranges^5^. HF is equivalent to the respiratory sinus arrhythmia and is considered to represent vagal control of heart rate^15^. Both vagal and sympathetic activities jointly contribute to LF HRV^16^. Normalized LF (LF%) and the ratio LF/HF are considered to mirror the sympatho-vagal balance or to reflect sympathetic modulations^5^. Reduced HRV has been established as a significant risk factor for higher mortality and CV death in ESRD population^4^,^17^,^18^. Hayano *et al.* had analyzed the association between HRV and mortality in 31 HD patients and found low HRV triangular index was associated with increased risk for both mortality and sudden death^17^. Longenecker *et al.* had also investigated the association between HRV and atherosclerotic CV disease in 115 chronic HD patients. They found low HRV parameters, including time domain measure of standard deviation of all R-wave-to-R-wave intervals (SDNN), standard deviation of all 5-min averaged intervals (SDANN) and HRV triangular index, were strongly associated with prevalent atherosclerotic CV disease^18^. Oikawa *et al.* had reported that a low SDNN value was related with both overall and CV mortality in 383 HD patients^4^. In addition, frequency-domain measures of HRV, including LF, HF and LF/HF, were lower in patients who died^4^. Consistent with previous findings, we found that HRV parameters, except HF%, were lower in patients with mortality than patients with survival, although the difference did not reach significant level, which may be related to small number of deceased patients.

The first major finding of our study was the identification of ΔHRV parameter as a risk factor for overall and CV mortality in patients with HD. Furthermore, adding ΔHRV parameter to the model consisting of various clinical parameters provided an additional benefit in the prediction of poor outcomes. Zitt E *et al.* evaluated the association of diabetes on autonomic CV regulation during HD. In contrast to 8 diabetic patients who showed a blunted autonomic response, in 9 non-diabetic patients, LF, HF and LF/HF increased during dialysis^19^. Impaired autonomic function might be related to diabetic autonomic neuropathy. Our recent study also found some HRV parameters (HF, LF%, LF/HF) increased after HD in patients without peripheral artery disease (PAD), but not in patients with PAD^20^. Changes in HRV could represent a compensatory mechanism for vascular arteriolar vasodilator capacity in patients without PAD, but diminished HRV in patients with PAD was caused by impaired sympatho-vagal equilibrium. In this study, VLF, LF% and LF/HF significantly increased after HD in patients with survival, whereas no HRV parameter increased significantly in patients with mortality. We propose that the obviously diminished compensatory increase in sympathetic and parasympathetic activity *per se* might enhance the risk for hemodynamic instability, and further related to overall and CV mortality in this study. Hence, an assessment of HRV change before and after HD in patients with HD may help identify the high-risk group with adverse outcomes.

The second major finding of our study was that ΔLF% was useful and stronger than HRV parameters before HD in predicting overall and CV mortality in HD patients. LF component of HRV correlates with the interaction of baroreflexes with peripheral vasomotor activity via the parasympathetic and sympathetic system. Previous studies have reported that low LF before HD was associated with adverse CV outcomes^4^, and LF increased after HD procedure^6^. Ferrario *et al.* had investigated the role of central volume in LF oscillations of HRV in HD patients. They found patients with low hydration status before HD showed an increased in LF after HD, whereas no significant change in LF was seen in high hydration status before HD^21^. In patients with lower hydration status, a reduction in central volume due to dialysis leads to a reaction of the capacity of the CV system to oscillate LF. However, for patients with higher hydration status, the same amount of fluid removal during HD does not reduce the central volume sufficiently to reactivate the oscillatory frequency. Barnas *et al.*^22^ had also evaluated the change in autonomic nervous system during HD. They found an increase in LF during non-hypotensive dialysis, but decreased during hypotensive dialysis. In Genovesi study, LF started from a lower baseline and increased progressively during HD procedure, indicating an increase of oscillatory sympathetic modulation during hemodynamic stable HD procedure^6^. The lack of sympathetic activation would lead to hemodynamic imbalance during unstable HD procedure. This implied that HD patients could maintain a hemodynamic stability during HD while sympathetic activation is induced. The proposed mechanisms responsible for changes in CV variability could be decreased oxidative stress, central volume status, baroreflex arch information or increased cardiac cholinergic responsiveness^21^,^23^. In our study, ΔHRV parameter was stronger than HRV parameters before HD in predicting adverse outcomes. Hence, the dynamic change of HRV before and after HD could reflect impaired CV autonomic neuropathy more accurately than resting HRV before HD can.

There are certain limitations in this study. First, there is a circadian pattern of heart rate autonomic modulation with a reduced HRV during the day because of increased sympathetic activity, and an increased HRV during the night due to the predominance of vagal modulation^24^,^25^. In our study, we performed all HRV examinations during the day (between 8 a.m. and 5 p.m.) to minimize the influence of circadian rhythm, but we could not eliminate the possibility that the rhythm might have been lost in some patients and the influence could not be defined. Perhaps a longer ECG record might help establish a baseline for each patient. In addition, HRV could be measured in the time or frequency domain. In our study, there was no time-domain measure. However, frequency-domain parameters correlated well (*r* = 0.85) with time-domain parameters, several time-domain parameters had proven to be useful for clinical purposes^26^,^27^. Finally, because of the relatively small numbers of mortality, the study statistical power had been reduced.

In conclusion, our results demonstrated that HRV parameters change before and after HD is useful and stronger than HRV parameters before HD in predicting overall and CV mortality, and it may offer an additional prognostic benefit over conventional clinical factors in patients with HD.

## Additional Information

**How to cite this article**: Chen, S.-C. *et al.* Heart Rate Variability Change Before and After Hemodialysis is Associated with Overall and Cardiovascular Mortality in Hemodialysis. *Sci. Rep.* **6**, 20597; doi: 10.1038/srep20597 (2016).

## Figures and Tables

**Table 1:** Comparison of baseline characteristics between patients with survival and with mortality.

**Characteristics**	**All patients (n = 182)**
Age (y/o)	61.2 ± 11.3
Male gender (%)	44.5
Smoking history (%)	25.8
Duration of dialysis (years)	5.9 (2.3–10.2)
Diabetes mellitus (%)	46.2
Hypertension (%)	62.6
Coronary artery disease (%)	27.5
Cerebrovascular disease (%)	16.5
Systolic blood pressure (mmHg)	152.7 ± 26.5
Diastolic blood pressure (mmHg)	81.6 ± 14.3
Laboratory parameters	
Albumin (g/dL)	3.8 ± 0.3
Fasting glucose (mg/dL)	119.2 ± 51.8
Triglyceride (mg/dL)	131 (92–210)
Total cholesterol (mg/dL)	182.5 ± 42.0
Hemoglobin (g/dL)	10.2 ± 1.1
Creatinine (mg/dL)	9.7 ± 2.1
Potassium (mEq/L)	4.5 ± 0.7
Total calcium (mg/dL)	9.4 ± 1.0
Phosphorous (mg/dL)	4.5 ± 1.0
Ca × P product (mg^2^/dL^2^)	41.8 ± 10.9
iPTH (pg/mL)	350.9 (186.5–479.6)
Kt/V	1.6 ± 0.3
Ultrafiltration/body weight (%)	4.2 ± 1.4
HRV parameters (frequency domain)	
before HD	
VLF (ms^2^)	4.06 ± 0.12
LF (ms^2^)	2.28 ± 0.28
HF (ms)	2.21 ± 0.32
LF% (nu)	39.00 ± 1.66
HF% (nu)	33.68 ± 1.19
LF/HF	0.08 ± 0.09

Abbreviations: Ca × P product, Calcium-phosphorous product; iPTH, intact parathyroid hormone; HRV, heart rate variability; VLF, very low frequency; LF, low frequency; HF, high frequency.

**Table 2:** HRV parameters of patients with survival and with mortality before and after hemodialysis.

**HRV parameters (frequency domain)**	**Survival**		**Mortality**	
	**Before hemodialysis**	**After hemodialysis**	**Before hemodialysis**	**After hemodialysis**
VLF (ms^2^)	4.07 ± 0.12	4.61 ± 0.13^**^	3.99 ± 0.34	3.96 ± 1.04
LF (ms^2^)	2.34 ± 0.28	2.84 ± 0.38	2.00 ± 1.01	2.53 ± 1.19
HF (ms^2^)	2.25 ± 0.31	2.35 ± 0.42	1.97 ± 1.13	3.53 ± 0.46
LF% (nu)	39.13 ± 1.80	47.28 ± 1.80^**^	38.33 ± 4.37	35.51 ± 4.15
HF% (nu)	33.53 ± 1.30	29.61 ± 1.16^*^	34.46 ± 3.02	31.35 ± 2.30
LF/HF	0.09 ± 0.10	0.48 ± 0.09^**^	0.02 ± 0.25	-0.07 ± 0.19

^*^p < 0.05, ^**^p < 0.001 com*p*ared to patients before hemodialysis.

Abbreviations are the same as in Table 1.

**Table 3:** Predictors for overall and cardiovascular mortality using Cox proportional hazards model.

**Outcomes**	**Multivariate**	
	**Hazard Ratios (95% CI)**	***p***
Overall mortality		
Age (per 1 year)	1.070 (1.021–1.122)	0.004
Albumin (per 1 g/dL)	0.088 (0.023–0.342)	<0.001
Kt/V (per 1)	0.139 (0.021–0.911)	0.040
Ultrafiltration (per 1%)	1.627 (1.147–2.308)	0.006
ΔLF % (per 1 nu)	0.978 (0.961–0.996)	0.019
Cardiovascular mortality		
Diabetes mellitus	4.084 (1.132–14.730)	0.032
Hemoglobin (per 1 g/dL)	0.410 (0.217–0.775)	0.006
ΔLF % (per 1 nu)	0.941 (0.914–0.970)	<0.001

Values expressed as Hazard Ratios and 95% confidence interval (CI). Abbreviations are the same as in Table 1.
